# Dietary Selenium Intake and Type-2 Diabetes: A Cross-Sectional Population-Based Study on CUME Project

**DOI:** 10.3389/fnut.2021.678648

**Published:** 2021-05-28

**Authors:** João Pedro Viana Dias, Paulo de Souza Costa Sobrinho, Adriano Marçal Pimenta, Helen Hermana Miranda Hermsdorff, Josefina Bressan, Luciana Neri Nobre

**Affiliations:** ^1^Faculty of Biological and Health Sciences, Department of Nutrition, Universidade Federal dos Vales do Jequitinhonha e Mucuri, Diamantina, Brazil; ^2^School of Nursing Department of Maternal and Child Nursing and Public Health, Universidade Federal de Minas Gerais, Belo Horizonte, Brazil; ^3^Department of Nutrition and Health, Universidade Federal de Viçosa, Viçosa, Brazil

**Keywords:** type 2 diabetes, selenium, food intake, cross-sectional study, adults

## Abstract

**Background and Aim:** Previous studies have suggested that the specific association between selenium (Se) and diabetes remains unclear. This study aimed to investigate the association between dietary Se and type-2 diabetes (T2D) in the Brazilian cohort [Cohort of Universities of Minas Gerais (CUME)].

**Methods and Results:** This cross-sectional study was conducted with a large sample comprising 4,106 participants of the CUME project, a concurrent open cohort restricted to a highly educated population group, composed of graduates of federal institutions of higher education located in the State of Minas Gerais, Brazil. Data on socioeconomic and dietary characteristics, as well as anthropometric measures, were collected from each subject for analysis. The sample was classified into energy-adjusted tertiles of dietary Se intake (μg/day). Differences in the continuous data were evaluated by the Kruskal–Wallis *H*-test (abnormal data), and the χ^2^-test assessed differences in qualitative data. As there was no relationship between T2D and Se intake in the bivariate analysis, multivariate analysis was not performed. The prevalence of T2D in the studied population was 2.8%. The mean age was 36 years. Regarding gender, 1,209 are males and 2,807 are females. Among females, the mean Se intake was 165.12 μg/day and the mean intake was 157.4 μg/day. Among males, it was 168.4 μg/day. Significant differences were observed across all Se intake tertiles in terms of age, gender, activity level, alcohol intake, energy intake, sugar, carbohydrates, lipids, fiber, and energy-adjusted meat intake. However, no significant differences were observed across all Se intake tertiles in terms of BMI, smoking status, and T2D. The results indicated that there was no significant association between dietary Se intake and the prevalence of T2D.

**Conclusion:** Dietary Se intake was not associated with the prevalence of T2D, despite the high intake of this micronutrient in the sample. These results contradict studies that identified the association between Se intake and T2D, with values of Se intake much lower than those observed in this study. Thus, this relationship seems to remain controversial.

## Introduction

Diabetes is a metabolic disorder that results from an insulin-production deficiency or its action, having hyperglycemia as one of its main signs (1). According to the International Diabetes Federation (IDF), in 2019, about 463 million adults aged between 20 and 79 years were diagnosed with this disease in the world, of which 15.5 million cases occurred in Brazil, which is the 5th country in the world regarding the number of diabetes cases in this age group (2).

Of the diabetes cases, about 90% corresponds to type-2 diabetes (T2D) mellitus (2), which is a multicausal disease mainly related to genetic factors and lifestyle, such as being overweight or obese; sedentary lifestyle; diets low in whole grains, fruits, nuts, and seeds; and diets rich in red meat, sugary drinks, and processed meat (3). Hyperglycemia, a characteristic of diabetes, is associated with acute and chronic complications of this disease, negatively affects the quality of life, and has a higher mortality rate in people with diabetes (1). Furthermore, hyperglycemia has been reported to cause an increase in reactive oxygen species production, causing oxidative stress and impacting the progression of T2D (4).

Antioxidant nutrients play an important role in the defense of our body by reducing the oxidative stress and preventing the emergence of chronic diseases, mainly by neutralizing free radicals and their metabolic effects. Vitamins A, C, and E and minerals, such as zinc, selenium (Se), copper, and manganese, which are present in our diet, are among the nutrients related to antioxidant status (5).

Selenium, one of the essential nutrients for humans and animals with the greatest antioxidant potential, is both organic and inorganic in nature (6). Its inorganic form, selenite salts and selenate, mainly accumulates in plants *via* the sulfur assimilation pathway. Plants absorb these salts from soil and convert them into the organic form, selenomethionine and selenocysteine, which may be incorporated into proteins, originating from selenoproteins (6). Animals and humans cannot synthesize these components and must ingest them as part of their diet (7).

The concentration of Se in plants is directly related to plant species, concentration in soil, type of soil, accumulation capacity, pH, salinity, organic matter, and redox reactions (7). Thus, Se dietary intake varies among countries and regions, and the type of food consumed. In plant tissues, its concentration depends on the geographic area and its level and availability in the soil (7). In animal tissues, it depends on the amount ingested (6).

Foods rich in selenium are meat, cereals, grains, and dairy products. Meat is the main source consumed, since skeletal muscle is the major site of selenium storage, accounting for ~28–46% of the total selenium pool (8).

Although animal-based foods are the best source of selenium, the Brazil nut (*Berthlletia excelsa*), a plant-based food, stands out as an exception, as it is the richest known source of selenium. The Brazil nut, which is part of the oilseed group, together with walnuts, peanuts, and cashew nuts, has a higher concentration of selenium. However, the concentration of selenium in Brazil nut varies considerably, depending on the geographic region of cultivation and the ability of the plant to absorb the mineral. Cardoso et al. (9) cite concentrations of selenium in Brazil nuts grown in São Paulo, Maranhão, Pará, and Amazonas ranging from 5 to 71.5 μg/g, while Silva Junior et al. (10) identified values ranging from 0.5 to 98 μg/g in Brazil nuts from Acre, Amapá, Amazonas, and Mato Grosso, which are states located in the north, southeast, and midwest regions of Brazil. This wide range among the levels of Se in Brazil nuts is explained by the geographic region where they were grown, and the capacity of each plant to absorb the mineral.

Silva Junior et al. (10) identified that the consumption of Brazil nuts grown in some Brazilian states (Amazonas and Amapá) exceeds the recommended intake value. In contrast, in other states (Acre and Mato Grosso), it does not even offer 10% of the selenium value recommended for consumption. These results demonstrate that it is difficult to define a single number of Brazil nuts to be consumed daily to achieve daily selenium consumption recommendations.

The selenoprotein family consists of 25 eukaryotic genes, with 25 human genes. All of these proteins have selenocysteine residue in their primary predefined structure (11). These selenoproteins are responsible for the function and regulation of thyroid hormones (4, 12), glucose metabolism (12), male fertility improvement (6, 12), and anti-inflammatory actions (6). They also indirectly participate in the mechanism of wound healing as oxidative stress reducers through glutathione peroxidase (GPX). GPX is the major selenoprotein present in the human body, and it assists in the control of excessive production of free radicals at the site of inflammation (6).

Recent research has focused on the relationship between selenium (Se) levels and glucose metabolism. Some observational studies have identified an association between high Se intake and a higher risk for T2D (4, 13, 14). Two meta-analyses (15, 16) have also reported this positive relationship. According to Ogawa-Wong et al. (17), the relationship between Se and T2D is a U-shaped curve, i.e., the onset of T2D occurs with insufficient or very high levels of Se. This finding shows the need to control Se intake so that it is neither scarce nor excessive.

Despite these findings, in the meta-analysis by Kohler et al. (16), the authors have reported that this relationship was found in observational studies and not in clinical trials. Thus, it is still not clear whether these differences are the result of uncontrolled misperception in observational studies or if there is a modest effect of Se and risk of T2D that may vary according to the length of exposure.

However, Vincet et al. (15) have reported that in non-experimental studies, a direct relationship between exposure to Se and risk of diabetes was identified, with a clear and almost linear trend in individuals with plasma or serum Se levels of above 140 μg/L when compared with the reference category of exposure to Se, which is 45 μg/L. A dose–response meta-analysis that focused on studies with direct assessment of dietary Se intake showed a similar trend. In experimental studies, it was shown that Se supplementation increased the risk of diabetes by 11% compared with participants allocated with placebo, regardless of gender.

In this sense, epidemiological studies on Se intake by the Brazilian population have identified that the Se daily intake has been above the recommended daily intake (RDI). The Se RDI for adults is 55 μg/day with a tolerable upper intake level (UL) of 400 μg/day (18). The Se intake values in a cohort of Brazilian adults, the ELSA study, identified a mean intake of 222 μg/day, being higher among women (19). The National Food Survey of the Family Budget Survey (FBS) (2008–2009) identified a mean intake value of 107.61 μg/day in the population, being higher among male adults (20).

However, research with smaller samples and residents of a single municipality, such as São Paulo, Teresina, and Manaus (21–23), has had different results. In these studies, among other parameters, the mean value of selenium intake and its biochemical parameters were evaluated, and the mean intakes were 41, 61, and 72 μg/day and erythrocyte values were 56.7 and 211 μg/L. Intake values were closer to those of the recommended. Regarding erythrocytes, in the first two studies, they were below the reference values (90–190 μg/L) (24), therefore showing that not all of the selenium ingested is absorbed. According to Soares (23), the mean selenium intake in the Brazilian population is very diverse (30 to 200 μg/day). It depends on the amount of Se in the soil, with foods from the north and northeast regions being richer in Se when compared to other Brazilian regions. However, we have not seen studies that evaluated the association of Se with diabetes in Brazilian populations.

Thus, this study aimed to investigate the association between dietary Se and T2D in a Brazilian cohort (CUME).

## Subjects and Methods

### Design and Study Population

This is a cross-sectional study with participants from the CUME project of two collection waves (March and August 2016, and between March and July 2018) in a virtual environment. In these two collection periods, 4,987 graduates answered the online survey questionnaire.

The CUME project is an open cohort with graduates from federal institutions of higher education in Minas Gerais, Brazil. The objective of the cohort is to assess the impact of specific food groups, nutrients, and dietary factors, and the nutritional transition on non-communicable diseases (NCD). The design, dissemination strategies employed, and project baseline profile have already been detailed in a previous publication (25). The CUME project was approved by the Human Research Ethics Committees of *Universidade Federal de Viçosa* (UFV) and *Universidade Federal de Minas Gerais* (UFMG) (Protocol No. 596,741-0/2013).

In this study, we did not include participants with incomplete questionnaires regarding demographic data (*n* = 531), from other nationalities (*n* = 22), Brazilians living abroad (*n* = 173), and likely type 1 diabetes: she/he was not diagnosed with diabetes in adulthood and uses insulin (*n* = 13). Participants with energy consumption below 500 kcal (*n* = 2) and above 6,000 kcal (*n* = 128) (26), meat intake >600 g/day (*n* = 184), nuts intake >100 g/day (*n* = 91) were also excluded from the study. Thus, the study sample consisted of 4,016 adults who graduated from the referred institutions and answered the 2016 and 2018 baseline CUME questionnaires.

### The Study Protocol and Data Collection

Invitations to participate in the research were sent by email to all graduates (graduates and postgraduates) from UFMG and UFV trained in the periods mentioned previously. The email addresses used were those in the Alumni Associations (UFV) databases and the Universities Technology and Information Directorates (UFMG).

For data collection, we used the self-administered baseline online questionnaire (Q_0), which was divided into two parts (accessed at http://www.projetocume.com/questionario). The first part consisted of questions related to sociodemographic and economic characteristics, lifestyle, individual and family referred morbidity, medication use, personal history of clinical and biochemical exams from the last 2 years, and anthropometric data. The second part of the questionnaire was sent a week after completion of the first part and had a quantitative Food Frequency Questionnaire (FFQ) composed of 144 food items, based on an original version previously validated in Brazil (27). As we did some modifications in the original FFQ, a validation study was also developed with a subsample of 146 CUME participants. The results showed a moderate agreement between the self-reported data and those directly measured from 24-h food recalls by telephone (overall intraclass correlation coefficient = 0.44, unpublished results).

### Evaluation of Variates

The outcome variable used for the analyses in this article was T2D, based on self-reported data from having a confirmed T2D diagnosis in adulthood and/or having blood glucose above 126 mg/dl in the previous year (1) and/or using oral antidiabetic and/or using insulin.

The exposure variable “selenium (Se) intake” was based on self-reported data on food intake; and subsequently, Se intake (μg/day) was calculated. Daily Se intake was adjusted by caloric intake using the residual method (28) and analyzed according to intake tertile.

Sociodemographic, lifestyle, and food intake were used as adjustment variables. Sociodemographic variables were gender (male, female) and age (years, continuous). Regarding lifestyle variables, smoking status (never smoked, former smoker, and current smoker), alcoholic beverage intake (never or does not consume, consumes), and physical activity (performs or does not perform scheduled physical activities) were assessed.

Food intake data were obtained from a quantitative FFQ. To minimize errors in the data collection process, images of food items and utensils were made available to facilitate the estimation of portion size and filling in of the report, and to obtain a more reliable response regarding the intake of the participants (29). There was a list of items that constituted the food group at the beginning of each page of the questionnaire. The participants were instructed to select the foods consumed in the previous year. For each food chosen, the participants indicated the portion size expressed in homemade measures commonly used in Brazil (teaspoon, tablespoon, ladle, knife tip, tongs, saucer, cup, and glass) or in traditional portions (unit, slices, and pieces) and the usual frequency of intake (day/week/month/year).

Consumption frequency of each food was transformed into daily consumption. Subsequently, daily consumption (grams or milliliters) was calculated by multiplying the portion size by the frequency of consumption. For the calculation of caloric intake (kcal) and nutrients, Brazilian tables of the nutritional composition of foods were used (30) and, if necessary, the table of the United States Department of Agriculture was also used (31).

For food consumption, the following were evaluated: energy intake, protein, meat, total lipids, animal fats, the relationship between saturated and polyunsaturated fatty acids, carbohydrates, sugars, fibers, and alcoholic drinks. For sugars, intake was quantified in grams of table sugar, brown sugar, honey, sweet treats, and soft drinks. Dietary variables were studied as a continuous variable and adjusted for energy using the residual method (28).

### Analysis of Results and Statistics

Sample characteristics are expressed as absolute and relative frequencies, or median and interquartile interval (percentile 25 to percentile 75), according to the medical diagnosis of T2D and sociodemographic, anthropometric variables, lifestyle, and food intake. Differences between continuous variables and categorized according to the presence or absence of T2D were assessed by the Mann–Whitney (abnormal data) test, and the χ^2^-test was performed to evaluate differences in qualitative data. Analysis of the prevalence of T2D according to the tertile of Se intake adjusted for energy and adjustment variables was carried out using the Kruskal–Wallis *H*-test (non-parametric data) χ^2^-test for categorized data. Hierarchical cluster analysis was carried out to group the Se intake using the centroid method to calculate Euclidean distances. All analyses were conducted using the SPSS statistical software, version 18.0, considering a significance level of 5%.

## Results

Of the total participants, 2.8% (*n* = 112) had T2D at the cohort baseline. The median energy-adjusted Se intake was 143.5 μg/day, and it did not differ with the intake of those with or without diabetes. The median, not energy-adjusted Se intake was 137 μg/day, and the average consumption was higher among men (148.9 μg/day) than among women (131.4 μg/day).

The food groups consumed by the participants that most contributed to selenium intake were meat and meat products (58.2%), starch (26.2%), dairy (8.2%), and oilseed, presented in the FFQ as Brazil nuts, walnuts, peanuts, and cashew nuts (7.5%). Age, gender, BMI, smoking cigarettes, and sugar intake differed statistically (*p* < 0.05) between the participants with and without T2D (Table 1).

**Table 1:** Characteristics of the study population according to type-2 diabetes status (*n* = 4,016).

**Characteristics**	**With T2D**	**Without T2D**	***p*-values**
N	112	3,904	
Age (years)^*^	42 (34; 53)^£^	34 (29; 40)	**<0.001**
Females (%)	64 (57.1)	2,743 (70.3)	**0.003**
Males (%)	48 (42.9)	1,161 (29.7)	
BMI (Kg/m^2^)	29 (26–34)^≠^	24 (21–27)	**<0.001**
Performs physical activity regularly (%)			
No	36 (32.1)	965 (24.7)	0.073
Yes	76 (67.9)	2,939 (75.3)	
Smokes cigarettes (%)			
Never smoked	80 (71.4)	3,128 (80.1)	**0.024**
Smoker or former smoker	32 (28.6)	776 (19.9)	
Drinks alcoholic beverages (%)			
No	32 (28.6)	1,072 (27.4)	0.795
Yes	80 (71.4)	2,832 (72.6)	
Energy intake (Kcal/day)	2,342 (1,690; 3,206)^£^	2,168 (1,691; 2,706)	0.052
Energy-adjusted proteins (g/day)	101 (89; 117)^£^	98 (86; 113)	0.097
Energy-adjusted meat (g/day)	207 (156; 287)^£^	194 (144; 258)	0.076
Total energy-adjusted lipids (g/day)	89 (75; 98)^£^	85 (75; 95)	0.176
Animal fat adjusted for energy (g/day)	32 (35; 37)^£^	30 (25; 35)	0.139
SFA/PUFA^**^ adjusted by energy	0.6 (0.5; 0.8)^£^	0.6 (0.5; 0.7)	0.337
Energy-adjusted carbohydrates (g/day)	260 (233; 299)^£^	273 (243; 303)	0.052
Energy-adjusted sugars (g/day)	6 (−2.2; 12.2)^£^	11 (5.5; 19.7)	**<0.001**
Energy-adjusted fibers (g/day)	29 (24; 35)^£^	29 (24; 34)	0.512
Energy-adjusted alcoholic beverage (g/day)	2.8 (0.8; 8.9)^£^	3.1 (1.0; 7.8)	0.959
Energy-adjusted selenium (μg/day)^*^	142 (121; 184)^£^	145 (121; 183)	0.661

* *Median*.

** *SFA, saturated fatty acid; PUFA, polyunsaturated fatty acid*;

£ *25 and 75 percentile*.

≠ *Minimum and maximum. The difference between continuous variables was assessed using the Mann–Whitney test; the χ^2^-test evaluated differences in the categorized data. Bold values mean they are statistically significant*.

The characteristics of the participants according to daily energy-adjusted Se intake, as well as sociodemographic, lifestyle, and dietary characteristics are shown in Table 2. Age, gender, physical activity, alcoholic beverage intake, and all the dietary variables studied differed according to tertiles of Se intake (*p* < 0.05). On the other hand, selenium intake was not associated with the presence of diabetes.

**Table 2:** Characteristics of the study population according to dietary selenium intake (*n* = 4,016).

**Characteristics**	**Selenium intake tertile**			
	**1st tertile (≤ 129.2 μg/day)**	**2nd tertile (> 129.2 <164.2 μg/day)**	**3rd tertile (≥ 164.2 μg/day)**	***p-*value**
Energy-adjusted Se intake^*^	109.6 (92.2; 120.8)	144.5 (136.9; 153.2)	220.2 (183.0; 320.2)	**<0.001**
Age (years)^*^	34 (29; 41)	33 (29; 39)	34 (30; 42)	**<0.001**
BMI (Kg/m^2^)	24.0 (21.5–27.3)^£^	23.7 (21.4–26.7)	24.1 (21.6–26.8)	0.154
Females (%)	875 (65.4)	959 (71.6)	973 (72.7)	**<0.001**
Males (%)	463 (34.6)	380 (28.4)	366 (27.3)	
Performs physical activity regularly (%)				
Yes	932 (69.7)	998 (74.5)	1,085 (81.0)	**<0.001**
No	406 (30.3)	341 (25.5)	254 (19.0)	
Smokes cigarettes (%)				
Never smoked	1,044 (78.0)	1,095 (81.8)	1,069 (79.8)	0.053
Smoker or former smoker	294 (22.0)	244 (18.2)	270 (20.2)	
Uses alcoholic beverages (%)				
No	413 (39.9)	355 (26.5)	336 (25.1)	**0.002**
Yes	925 (69.1)	984 (73.5)	1,003 (74.9)	
Energy intake (Kcal/day)	2,384 (1,876; 2,984)^£^	1,861 (1,475; 2,352)	2,241 (1,817; 2,824)	**<0.001**
Energy-adjusted proteins (g/day)	89 (78; 100)^£^	102 (93–114)	106 (90; 124)	**<0.001**
Energy-adjusted meat (g/day)	155 (110; 205)^£^	209 (167; 262)	226 (159; 311)	**<0.001**
Total energy-adjusted lipids (g/day)	81 (68–93)^£^	84 (76–93)	90 (80–101)	**<0.001**
Energy-adjusted animal fat (g/day)	29 (24; 35)^£^	30 (27; 35)	30 (26; 35)	**<0.001**
SFA/PUFA^**^ ratio adjusted by energy	0.7 (0.5;0.8)^£^	0.6 (0.6; 0.7)	0.5 (0.5; 0.6)	**<0.001**
Energy-adjusted carbohydrates (g/day)	295 (266–327)^£^	272 (249–297)	256 (224; 282)	**<0.001**
Energy-adjusted sugars (g/day)	14 (6; 25)^£^	12 (7; 19)	8 (4; 15)	**<0.001**
Energy-adjusted fibers (g/day)	31 (24; 38)^£^	28 (23; 32)	29 (24; 34)	**<0.001**
Energy-adjusted alcoholic beverage (g/day)	2.5 (0.3; 7.3)^£^	3.4 (1.7; 7.8)	3.4 (0.9; 8.2)	**<0.001**
Type-2 diabetes status				
Yes (%)	39 (2.9)	36 (2.7)	37 (2.8)	0.936
No (%)	1,299 (97.1)	1,303 (97.3)	1,302 (97.2)	

* *Median*.

** *SFA, saturated fatty acid; PUFA, polyunsaturated fatty acid*.

£ *25 and 75 percentiles. The difference between continuous variables was assessed using the Kruskal–Wallis H-test; the χ^2^-test evaluated differences between categorized data. Bold values mean they are statistically significant*.

**Figure 1:**
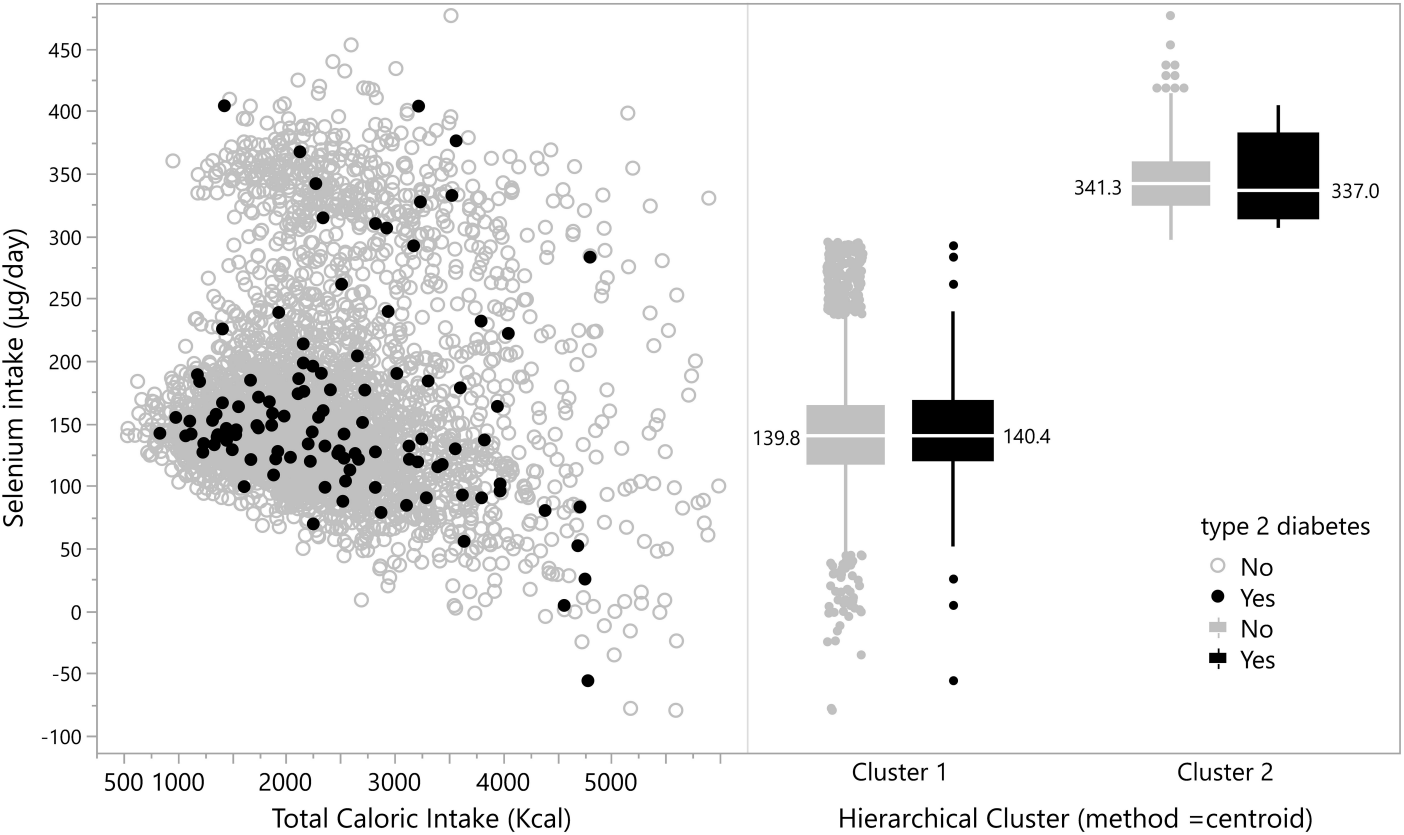
Distribution of participants according to energy-adjusted Se intake and T2D prevalence.

The distribution of the participants according to energy-adjusted Se intake and T2D prevalence is shown in Figure 1. Cluster analysis identified two distinct profiles of Se intake among the studied graduates, one with median intake of 139.8 μg/day and another with median intake of 341.3 μg/day (*p* < 0.05), but no significant association with T2D prevalence (*p* < 0.05) was seen.

**Figure 2:**
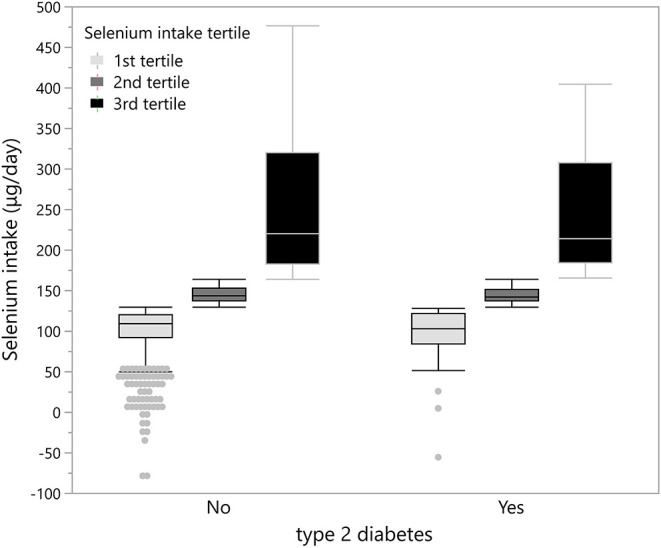
Distribution of participants according to tertile of energy-adjusted Se intake.

Figure 2 shows that both people without and with T2D have an average energy-adjusted Se intake at three very similar levels, which makes it very difficult to identify the relationship between Se intake and T2D among the participants in this study.

## Discussion

We conducted a cross-sectional study on a sample of 4,016 young adults from a cohort of public University graduates in Brazil, with the primary objective of investigating the association between dietary Se and T2D. The prevalence of T2D in the studied population was 2.8%, which is close to that estimated in a study with a representative sample of Brazilian adults in 2019 for people with over 12 years of schooling (3.5%) (32).

Although the study participants were still young, T2D was seen to be more prevalent among the elderly, confirming that the prevalence of T2D increases with advancing age. However, it is noteworthy that the effect of nutrients on health outcomes may require a long exposure time. Cohort studies are recommended for this evaluation. Thus, although the CUME project is a cohort study, this is a cross-sectional study. In the future, we will verify that a long time of exposure to high consumption of selenium may favor T2D. Furthermore, this study did not evaluate blood selenium biomarkers (such as plasma selenium and erythrocytes), which provide more reliable information on selenium intake and the body reserve of this mineral (33).

It is also noteworthy that not all selenium ingested is absorbed. The Se absorption mechanism differs between consumed sources, and this difference determines its absorption rate. Most selenite is absorbed in the duodenum by passive diffusion, while selenate is actively absorbed in the ileum by cotransport with sodium ions. Selenium from selenomethionine is also absorbed in the small intestine, with the highest absorption rate in the duodenum. Its absorption occurs through the sodium-dependent system and has the same mechanism as the methionine, as well as selenocysteine, which competes with cysteine, lysine, and arginine (34). Furthermore, the organism tries to maintain its homeostasis and generally has higher absorption when its reserves are reduced, and less absorption when inadequate or excessive (35).

The profile of the participants in this study was previously described by Gomes-Domingos et al. (25). It is a group made up predominantly of graduates from undergraduate and graduate courses at public universities in the state of Minas Gerais and residents of all Brazilian states and the federal district. Of the total participants, 72.9% has a postgraduate degree, are young adults with a mean age between 30 and 39 years (46.4%), and live on an individual income of up to five minimum wages (49.5%).

The characteristics of the participants according to T2D status are similar to the studies conducted by Wei et al. (14) and Stranger et al. (13) regarding difference in age and BMI of the participants, according to T2D status (*p* < 0.05). However, these studies (13, 14) identified differences in selenium and alcohol intake, consumption of energy, fibers, and animal protein (14) according to T2D status, a fact that was not observed in this study. Nevertheless, it is noteworthy that different from this research, the nutrient intake in these studies (13, 14) was not adjusted for energy consumption.

The results of this research did not identify a positive and significant association between energy-adjusted Se intake and the prevalence of T2D. Thus, our findings corroborate other studies that did not identify an association between dietary Se and T2D (36). Thompson et al. (36) found no significant difference between the appearance of T2D between the control group and the placebo from Se supplementation of 200 μg/day. As mentioned earlier, according to Ogawa-Wong et al. (17), the relationship between Se and diabetes is a U-shaped curve. That is, the onset of the disease occurs in situations of very low or very high levels of Se. In this study, the Se intake of the participants was within the acceptable range. It was above the RDI but below the tolerance limits for this mineral.

The relatively young age of the studied participants and the method used to assess selenium intake could justify the absence of an association between selenium intake and T2D in this research. However, some studies (11, 12, 35, 36) that identified a positive association between selenium intake and T2D were also carried out with young participants (30 to 52 years old), that used a frequency of consumption questionnaire to estimate intake selenium. And unlike the present study, which presented a consumption of selenium adjusted for energy well above the recommendation value (143.5 μg/day), selenium intake in these other studies was much closer to the recommendation value (11, 12, 35), or above (36), but much lower than that observed in our study. Therefore, indicates the need for further research to confirm the association between the consumption of selenium and type 2 diabetes.

According to Parekh et al. (37), an intake of Se between 50 and 400 μg/day is considered a safe range for adults, while 850–900 μg/day may reach a toxicity level. We carried out this analysis to verify whether the graduates who consumed below or above the values considered safe showed higher prevalence of T2D, and no significant association was observed. Among those with T2D, four participants consumed Se above 400 μg/day and one below 50 μg/day.

Although some epidemiological studies have explored the association between Se and T2D, the results are still inconclusive. Some researchers have cited that a high level of Se may reduce the prevalence of diabetes (38, 39). In contrast, others have mentioned that a high serum Se level may be related to the increased prevalence of diabetes (40, 41), or that there is no significant relationship between Se and risk of T2D (42).

In a randomized trial of Se supplementation, Stranges et al. (43) identified a significantly increased risk of T2D in participants who used supplemental Se. These findings are corroborated by a placebo-controlled trial conducted by Faghihi et al. (44), which identified that Se supplementation might be associated with adverse effects on blood glucose homeostasis in patients with T2D.

On the other hand, observational studies on dietary Se intake and diabetes have identified an association between high Se intake and higher risk for T2D. Stranges et al. (13), after monitoring 7,812 women in a 16-year cohort study in northern Italy, found an odds ratio of 1.29 (95% CI: 1.1, 1.52) for an increase of 10 μg/day in Se intake, associating with an increased risk for T2D. The association was also found in a study that assessed 19,931 North American individuals. The same increase in daily intake found in Italy led to a rise in the prevalence of T2D by 12% (OR: 1.12; CI: 95 %: 1.06–1.18) (4). Siddiqi et al. (45) also found a positive linear association in a population of 8,824 adults in Heilongjiang province in northern China. The dietary intake of Se was associated with an elevated risk of T2D in both genders. Wei et al. (14), who carried out a cross-sectional study with 5,423 middle-aged and elderly adults in Hunan province of China, also identified a positive and significant association between Se dietary intake and the prevalence of diabetes.

Meta-analyses published by Vincet et al. (15) and Kohler et al. (16) have reported that this relationship exists. According to Vincet et al. (15), in general, results of experimental and non-experimental studies indicate that Se may increase the risk of T2D in a wide range of exposure levels. The relative increase in risk is slight but of possible importance for public health because of the high incidence of diabetes and exposure to Se. However, according to Kohler (16), in the set of studies evaluated, the relationship between Se and T2D differs between observational studies and randomized clinical trials. This relationship is found in observational studies and not in clinical trials. In randomized clinical trials, a higher risk of T2D was not observed in those who received Se compared to placebo. Thus, it is still not clear whether these differences are the result of an uncontrolled misperception in observational studies or if there is a modest Se effect and T2D risk that may vary according to the length of exposure (16).

Studies on the relationship between Se and T2D have considered different sources: serum Se, nails, urine, diet, plasma, and erythrocyte. Other types of studies, such as cross-sectional, case control, and cohort, have divergent results. Cross-sectional studies with the American population (40, 41, 45) identified that high serum Se levels were positively associated with the prevalence of diabetes. A cohort study found that plasma Se was marginally significantly related to the occurrence of blood glucose changes in males but not in females (46). In contrast, other studies (38, 39) have identified that serum Se values were significantly lower in patients with T2D, or that Se in the diet was not associated with the development of disorders in glucose metabolism or diabetes (47). They have shown that Se supplementation was not associated with elevated plasma glucose levels (48).

It is important to highlight that in this research, selenium intake was estimated, and this may not reflect the status of body selenium. In addition, since the participants are young, perhaps a longer exposure time to high selenium values will be necessary for this outcome (T2D) to be evident. Nevertheless, some epidemiological research on the association between selenium and fT2D mentioned in this article also used an estimate of the selenium dietary intake assessed by means of a food record (13, 14, 45, 49).

Since Se has multiple effects on the human body, it may be both protective and a risk for T2D. According to Mueller et al. (50) and Steinbrenner et al. (51), its antidiabetic effect is due to its antioxidant capacity. However, its therapeutic range is relatively narrow. Some Se compounds may generate reactive oxygen species with toxic effects (39), and overaccumulation of these reactive species may increase insulin resistance and impair the function of pancreatic β cells (52). Furthermore, higher dietary Se intake may increase the release of glucagon and consequently increase hyperglycemia (53), and increase the expression of glutathione peroxidase 1. The high activity of this enzyme may interfere with insulin signaling, favoring resistance to the action of insulin, and hyperinsulinemia (54).

The main strength of this article lies in the fact that it is the first to assess the association between dietary Se intake and T2D in the Brazilian population. The studies mentioned here have been conducted in the United States, Europe, and China. It is important to assess this association, particularly for the Brazilian population, because of differences in ethnicity, geography, and eating habits, which may affect the results. It is important to know that Se is a very common nutrient in food, especially in Brazil nuts, known in the world as the food with the highest concentration of this nutrient (10). However, in this study, oilseeds were the food that least contributed to the Se ingested by the participants, and meat was the one that stood out as source of selenium. Although the frequency of consumption questionnaire is the most suitable for food consumption studies in population research, it may overestimate consumption, which is not recommended when the objective is to assess nutrient intake quantitatively (55).

Nevertheless, there are some limitations to this research. First, it is a cross-sectional study, which is not able to explain the causal relationship of an outcome; therefore, further prospective studies are needed to confirm the findings. Second, the information is self-reported in an online questionnaire, and the serum Se level has not been measured. Studying the relationship between dietary Se, serum Se, and diabetes may provide a more comprehensive understanding of this topic. On the other hand, the sample studied is extensive, including people from all Brazilian states, which allowed us to examine the association between dietary Se intake and diabetes, thus suggesting a reasonable representation of the Brazilian population in the study.

This study did not identify an association between Se intake and T2D from the studied sample. Thus, this relationship is seen to remain controversial, and further research is required, especially of the cohort type. In addition to selenium intake, blood biomarkers should also be evaluated.

## Data Availability Statement

The raw data supporting the conclusions of this article will be made available by the authors, without undue reservation.

## Ethics Statement

The studies involving human participants were reviewed and approved by Universidade Federal de Minas Gerais (UFMG) (Protocol No. 596,741-0/2013. The patients/participants provided their written informed consent to participate in this study.

## Author Contributions

LN and JD designed the study, interpreted the data, and wrote the manuscript. PC analyzed the data with supports by LN and AP. JB, HH, and AP built the database for the cohort. LN takes responsibility for the integrity of the data and accuracy of the data analysis. All authors have read the manuscript and took part in the discussion.

## Conflict of Interest

The authors declare that the research was conducted in the absence of any commercial or financial relationships that could be construed as a potential conflict of interest.
